# Elevated reproduction does not affect telomere dynamics and oxidative stress

**DOI:** 10.1007/s00265-016-2226-8

**Published:** 2016-10-07

**Authors:** Joanna Sudyka, Giulia Casasole, Joanna Rutkowska, Mariusz Cichoń

**Affiliations:** 1 Institute of Environmental Sciences, Jagiellonian University, ul. Gronostajowa 7, 30-387 Kraków, Poland; 2 Behavioral Ecology and Ecophysiology Group, Department of Biology, University of Antwerp, Universiteitsplein 1, 2610 Wilrijk, Belgium

**Keywords:** Ageing, Antioxidant, Oxidative damage, Cost of reproduction, Songbird, Low temperature

## Abstract

**Abstract:**

Oxidative stress and telomere dynamics are considered to be powerful biomarkers quantifying a potential trade-off between current reproduction and self-maintenance. Recent studies confirmed the negative impact of elevated reproduction on telomeres, but the evidence for the cost of reproduction in terms of oxidative stress remains equivocal. In order to induce reproductive costs, we experimentally manipulated reproductive effort by increasing brood size in captive zebra finches (*Taeniopygia guttata*) and additionally challenged all birds by a low ambient temperature to facilitate detection of these costs. We were not able to show any negative effects of elevated reproductive effort on telomere dynamics and oxidative stress among parents, although brood enlargement was effective in terms of total mass and number of fledged young. Interestingly, irrespective of brood size treatment, we found a significant increase in antioxidant capacity at peak breeding while oxidative damage did not change with time. Our results may suggest that reproduction, instead of generating costs, may stimulate physiological functions promoting self-maintenance in terms of higher protection against free radicals. Possibly, opportunistic breeders such as zebra finches may not impede their future performance for the sake of current reproduction.

**Significance statement:**

This study interrogates a molecular background behind one of the most intriguing trade-offs that potentially occurs between self-maintenance and reproduction. We manipulated breeding effort in zebra finches to understand if the cost of reproduction can be mediated by telomere dynamics and oxidative stress. In our study system, we did not detect the direct reproductive costs in terms of parental oxidative damage and telomere loss; instead, these costs were paid by the offspring in terms of their inhibited growth rate. Moreover, we found that entering into the reproductive state strongly stimulated self-maintenance by increasing antioxidant capacity in parents. Our results emphasize that current reproductive success is not always prioritized over investment in body maintenance preventing the oxidative cost of reproduction.

**Electronic supplementary material:**

The online version of this article (doi:10.1007/s00265-016-2226-8) contains supplementary material, which is available to authorized users.

## Introduction

The cost of reproduction is often detected in experimental studies in terms of diminished body condition and survival (Nur 1984a; Daan et al. 1996; Flatt 2011; Hayward et al. 2015), subsequent fecundity of parents and offspring and offspring survival (Gustafsson and Sutherland 1988; Deerenberg et al. 1996) and as such constitutes an important support for life-history theory. It is generally assumed that life-history trade-offs have to be driven by various physiological mechanisms; however, those mechanisms still remain equivocal. Telomere dynamics and oxidative stress could act as such potential mediators underlying a trade-off that should occur between reproduction and survival (Heidinger et al. 2012; Speakman et al. 2015). Oxidative stress potentially affects telomere dynamics (Von Zglinicki 2002), but both processes may independently mediate the cost of reproduction (Monaghan and Haussmann 2006; Metcalfe and Monaghan 2013). Oxidative stress is basically a displacement in the sensitive balance between the inevitable production of reactive oxygen species (ROS) and their neutralization via the antioxidant defence or oxidative damage repair systems. ROS, the by-products of oxidative phosphorylation in the mitochondria, have an important signalling role, but are mostly known for being extremely damaging to biomolecules: lipids, proteins and DNA. Compared with other regions of DNA, telomeres seem to be particularly subjected to ROS (Von Zglinicki 2002). Eroding telomeres are strongly involved in cellular and organismal ageing (Monaghan and Haussmann 2006) and seem to predict life expectancy (Bize et al. 2009) whereas accumulating oxidative damage alone may lead to accelerated ageing (Sohal and Weindruch 1996). Since reproductive activity is positively associated with the ageing phenotype, elucidating actuarial, survival-related senescence (Nur 1984a; Flatt 2011; Hayward et al. 2015), the idea arises that telomeres and oxidative stress may serve as biomarkers of reproduction-associated decreases in survival probability. Reproduction elevates metabolic rate (Deerenberg et al. 1995; Skibiel et al. 2013) due to higher workload (Nur 1984b; Tinbergen and Verhulst 2000), which potentially leads to overproduction of telomere-damaging ROS (Alonso-Alvarez et al. 2004; Wiersma et al. 2004). We hypothesize that resources potentially fuelling somatic maintenance (such as antioxidant production or telomere protection against ROS) could be redirected to cover increased energetic demands caused by reproduction, as expected when two life-history traits share a common resource pool (Zera and Harshman 2001). A diversion of resources maintaining oxidative status has already been demonstrated during reproduction (Beaulieu et al. 2015). If ROS are not neutralized, they could damage cells and precipitate cellular divisions resulting in further telomere loss (Von Zglinicki 2002).

Indeed, it has been already demonstrated that reproduction triggers telomere loss in both correlative (Kotrschal et al. 2007; Heidinger et al. 2012; Plot et al. 2012; Bauch et al. 2013) and experimental studies (Reichert et al. 2014; Sudyka et al. 2014). However, the link between oxidative stress and reproduction remains ambiguous. Detrimental effects of reproduction on oxidative balance have been shown in some studies (Bergeron et al. 2011; Heiss and Schoech 2012; Olsson et al. 2012), but others found no evidence that reproduction increases oxidative damage (Markó et al. 2011; Garratt et al. 2012). Interestingly, in recent studies, reproductive activity has been reported to reduce oxidative stress (Garratt et al. 2011; Ołdakowski et al. 2012, 2015; Costantini et al. 2014), and so the hypothesis that oxidative damage mediates the reproduction–lifespan trade-off seems not to be adequately supported. Such ambiguity may result from the fact that most studies do not manipulate reproductive effort directly, but compare reproducing vs non-reproducing animals instead (reviewed in Metcalfe and Monaghan 2013, but see Speakman and Garratt 2014). These discrepancies have been also accounted for by the oxidative shielding hypothesis, which suggests that reproduction-induced reduction in oxidative stress evolved to protect mothers and offspring from oxidative insults (Blount et al. 2015).

In this study, we experimentally elevated reproductive investment in captive zebra finches (*Taeniopygia guttata*) by means of brood size manipulation. We aimed at evaluating whether telomere dynamics and oxidative stress are affected by the level of reproductive effort and, in consequence, whether resulting changes in telomere length and oxidative status may elucidate reproductive costs. Since these costs are more likely to be detected under unfavourable breeding conditions (e.g. Descamps et al. 2009), we additionally constrained the birds by exposing them to a low ambient temperature. Telomere length (TL), oxidative damage compounds (dROMs) and antioxidant capacity (OXY) were quantified in blood. We expected greater shortening of telomeres and higher oxidative stress (increased oxidative damage and reduced antioxidant capacity) among parents experiencing experimentally elevated reproductive effort in comparison to control, non-manipulated birds.

## Materials and methods

### Study system and sampling

In January 2014, we randomly mated birds with unrelated partners and kept each breeding pair in a visually isolated cage (70 × 30 cm and 40 cm high), all placed in one climatic chamber. One day before mating, the climatic chamber was set at 10 °C. This is far below thermoneutral temperature for zebra finches, but it is commonly experienced by birds reproducing under field conditions (Zann 1996). During light periods (12 h:12 h, light:dark, light on at 8:00), the temperature reached 12 °C. This temperature is considered a mean minimum daily temperature required for breeding in wild zebra finches (Zann 1996). Thus, all experimental animals were facing an additional, energetically demanding burden, which is often recommended in studies concerning reproductive costs (Metcalfe and Monaghan 2013; Speakman and Garratt 2014). Trade-offs are usually easier to detect while animals experience unfavourable or fluctuating conditions (van de Crommenacker et al. 2011; Heiss and Schoech 2012; Fletcher et al. 2013). A lack of resource limitations may impede any trade-off, when the conflicting allocation is not necessary to cover all vital functions. The obvious way to limit resources would be to reduce food supply. However, constraining the amount of food has been shown to reduce investment in reproduction; hence, it is not appropriate to evaluate the effects of elevated reproductive effort on studied parameters (Speakman and Garratt 2014). Instead, it has been proposed that animals could be challenged with an additional burden to the energy budget, such as reduced ambient temperature.

In our study, the birds were fed ad libitum with a seed mixture dominated by different millet species (Megan, Poland) and supplemented with hard-boiled chicken eggs mixed with carrot, vitamins (C, A, B_1_, B_6_, B_12_, D_3_ and K; Ornitovit Kanarki, Dolfos, Poland) and mineral sand three times per week before hatching of their young. For 12 days after hatching, the egg/carrot mix was given five times per week. The birds were also provided with water and a cuttlebone.

Breeding attempts were monitored daily starting from the day of pairing. After hatching, broods with a similar hatching date and nestling number (±1) were matched in experimental pairs. Two days after hatching of the first chick, one randomly chosen nest in each pair was enlarged by two nestlings (ca. 40 % increase in brood size), originating from a donor nest (which provided various numbers of nestlings, from 1 to 6, mostly to more than one recipient nest), and with identical hatching dates as the nestlings from recipient broods. These donor nests were not included in further analyses. The other nest in a pair constituted a control nest with the brood size left non-manipulated. In addition, half of the nestlings (only original nestlings, excluding those from donor nests) within each experimental pair were swapped between the nests at the day of brood manipulation and all nestlings were uniquely marked by nail clipping. Cross-fostering was performed for the purpose of another study, but it was also beneficial by equalling environmental and handling stress between enlarged and control broods. If a nestling died within 1–2 days after hatching, we replaced it with another one coming from a donor nest to keep the brood size unchanged. Our experimental design did not include broods with reduced numbers of nestlings, because it would be more difficult to obtain a reasonable number of trios. More importantly, most studies employing brood size manipulation demonstrate that brood reductions usually do not bring about any significant effects in comparison to non-manipulated controls (Santos and Nakagawa 2012). We established 18 control (median number of chicks at the day of brood size manipulation 5, range 4–6) and 18 enlarged nests (median 7, range 6–8). All enlarged broods’ parents had to cope with more nestlings than they were prepared to in that current reproductive event. Such a manipulation has been repeatedly shown to increase reproductive effort and energy expenditure among parents attending enlarged broods (Deerenberg et al. 1995; Santos and Nakagawa 2012).

**Fig. 1:**
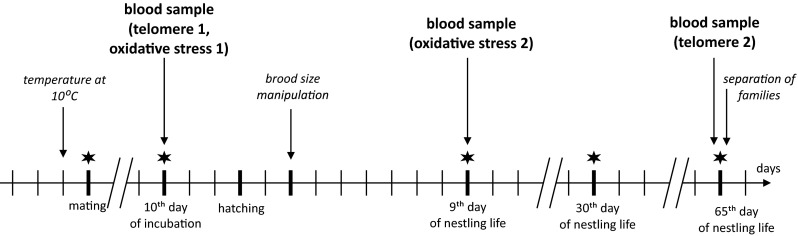
Timeline of the experiment. The difference in sampling points for telomeres and oxidative stress stems from the fact that changes in oxidative balance can be detected rapidly and represent a snapshot at our focal point, while changes in TL may only be visible after a period of time allowing for the turnover of blood cells. Body mass measurements of adults are marked by a *star*, and the *tick marks* represent 1-day intervals

We blood-sampled adults three times: (i) before the brood size manipulation (at the end of incubation); (ii) when nestlings were 9 days old (average per each nest), when the energetic cost of rearing nestlings (Lemon 1993) and the feeding rate (ten Cate 1982) are the greatest; and (iii) at the end of the experiment (65 days after hatching), when the offspring reached full independence (Fig. 1). The blood sample from each parent was placed in two capillaries (up to 75 μl per capillary, one for telomere and one for oxidative stress measurements) after a brachial vein puncture. Body mass of adults (Fig. 1) and nestlings (Fig. S1) was measured at five different time points to the nearest 0.01 g. The right tarsus of all birds was measured when nestlings were 30 days old to the nearest 0.01 mm. At the end of the experiment (65 days after hatching), nestlings and parents were separated and placed in outdoor aviaries. It was not possible to record data blind because our study involved focal animals in the experimental groups.

### Telomere analysis

Immediately after sampling, whole blood was placed in 96 % ethanol and stored at 4 °C. Subsequently, it was used for DNA extraction with the Blood Mini kit (A&A Biotechnology, Gdynia, Poland; modified for overnight incubation in 37 °C). The DNA concentration and purity was measured with a NanoDrop 1000 Spectrophotometer (ThermoScientific, Waltham, MA, USA) and the integrity of each sample was confirmed by an electrophoresis on a 1 % agarose gel.

TL was assessed by the real-time quantitative PCR assay adapted for birds (Criscuolo et al. 2009). We used relative TL, expressed as the ratio (T/S) of a telomere copy number (T) and a single control gene copy number [S, which was GAPDH, (Cawthon 2002)]. Five nanogrammes of DNA per reaction was used in qPCR for both sets of primers. GAPDH and telomere primers’ concentrations were 80 and 40 nM, respectively, for final volume of 10 μl per reaction well containing 5 μl of Brilliant III Ultra-Fast SYBR QPCR MM (Agilent Technologies, Santa Clara, CA, USA). Both amplicons (GAPDH and telomeres) were placed on one plate, along with a negative control for each. Telomere amplicon was run in triplicate, while GAPDH in duplicate. Both samples from one individual were assayed on the same plate to avoid between plate variation within individual, whereas experimental and control individuals were evenly and alternately distributed on a plate. If the variation between technical replicates (Ct SD) exceeded 0.5, both samples for each individual were measured again. To generate a standard curve for amplification efficiency, each plate included serial twofold dilutions of a reference DNA (mixed DNA of three birds not included in the study) run in duplicate form 10 to 0.16 ng for telomere and from 10 to 0.62 ng for GAPDH. The same DNA was used as the GOLDEN sample to account for inter-plate variation and run in triplicate on every plate. Mean amplification efficiency and the determination coefficient (*r* ^2^) of the standard curve were 95 % (range 88–102 %) and 0.989 (range 0.974–0.998) for GAPDH and 101 % (range 92–110 %) and 0.957 (range 0.941–0.982) for telomeres, respectively. Mean intra-plate SD for the *Ct* variance [repeatability (Bustin et al. 2009)] was 0.22 for telomeres and 0.12 for GAPDH, whereas inter-plate coefficient of variation (reproducibility, 100 × SD/mean value) and SD calculated on the GOLDEN sample’s T/S ratios were 12.01 % and 0.09 respectively. The CV of T/S ratios (not adjusted for the reference GOLDEN sample) between all individuals was 53.15 %, and SD of between-individual variation = 0.56. In total, we ran 20 plates. For further details on primers, reaction setup and T/S ratio calculation please refer to Sudyka et al. (2014). From further analyses, we had to exclude one control female since we missed the whole blood sample from the first sampling. As a result we analysed 71 individuals (35 females and 36 males). TL of the same individual was correlated between the two measurements (*r* = 0.389, *P* = 0.001 across individuals). This can be perceived as a lower bound estimate of repeatability of telomere measurements, as any variation may also be caused by the treatment effect.

### Oxidative stress analysis

To evaluate oxidative stress we measured oxidative damage compounds with the dROM and antioxidant capacity with the OXY-ADSORBENT (OXY) colorimetric tests (both Diacron International, Grosseto, Italy). The blood for oxidative stress analyses was centrifuged immediately after collection, then plasma was snap-frozen in liquid nitrogen and stored at −80 °C until analysis. We followed the protocols in Costantini et al. (2011).

The dROM test measures hydroperoxides (reactive oxygen metabolites—ROMs) that are compounds produced in the early phases of the oxidative cascade. In this test, the ROMs of the plasma, in the presence of iron, generate the alkoxyl (R–O·) and alkylperoxyl (R–OO·) radicals that are highly reactive. When these compounds oxidize an aromatic amine, contained in the chromogen, they produce a complex whose colour intensity is directly proportional to the plasma concentration of ROMs (Costantini and Dell’Omo 2006). The concentration of ROMs was calculated by comparison with a standard curve obtained by measuring the absorbance of a standard solution. The results of the d-ROMs test were expressed as mmol of H_2_O_2_ equivalents. The OXY test measures the total antioxidant capacity by quantifying the ability of the plasmatic antioxidant barrier to cope with the oxidant action of hypochlorous acid (HClO), which is an endogenously produced oxidant, relevant in biological systems. In this test, the oxidant solution is put “in excess”, compared to the adsorption ability of the sample. The residual HClO reacts with an alkyl-substituted aromatic amine solubilized in the chromogen, oxidizes it and transforms it into a pink derivate. The intensity of the coloured complex is inversely related to the antioxidant capacity (Costantini and Dell’Omo 2006). Values were expressed as mmol of HClO neutralized. For dROMs, mean CV between replicates was 3.63 % while inter-plate CV was 14.06 %, whereas for OXY, mean CV between replicates was 4.03 % and inter-plate CV was 8.94 %. Both samples from one individual were assayed on the same plate to avoid between plate variation within individual, whereas experimental and control individuals were evenly and alternately distributed on a plate. We could not measure OXY and dROMs in all individuals because we had not enough plasma for carrying out both analyses or the samples were haemolysed or impossible to analyse due to a high content of lipids in the plasma. In further analyses, we only used individuals that had measurements from both samplings. As a result, we analysed dROM measurements for 52 individuals (31 females and 21 males) and OXY for 65 individuals (36 females and 29 males). dROMs and OXY between the 1st and the 2nd sampling event were correlated within individual (*r* = 0.363, *P* = 0.008 and *r* = 0.248, *P* = 0.046, respectively, across individuals). This can be seen as a lower bound estimate of repeatability of oxidative stress measurements, as any variation may also be caused by the treatment effect.

### Statistical analyses

To confirm random assignment of individuals to the experimental groups, we compared their age and body mass at mating and TL, dROMs and OXY measured at the first blood sampling (the end of incubation–before the treatment), using linear mixed models (LMM) based on REML with brood size treatment, sex and age (not in age analysis) as explanatory variables. Age (in days of life) was defined as a continuous variable. To test whether elevated reproductive effort affects body mass, TL, dROMs and OXY, we employed LMM with a repeated measure design. We included brood size treatment, sex and time [five time points for body mass measurements, see Fig. 1; two time points for TL, dROMs and OXY: (i) before the brood size treatment and (ii) at a focal point for each, see Fig. 1)] as explanatory variables in a separate model for each dependent variable. We used a binominal generalized mixed model (GLMM) to test survival prospects in adults [including brood size treatment, sex, age and telomere length change (telomere2-telomere1) as fixed factors]. In all analyses, we checked interactions between the factors, but they were retained in the models and reported only if significant (*P* < 0.05), apart from the focal experimental treatment-by-time interaction, which is always reported as it constitutes our main prediction. In all analyses, we also considered random effects—nest id and individual id—in the case of repeated measure analyses, to avoid pseudoreplications. If any random parameter was redundant (variance was 0), it was removed from a model. In all analyses, we tested for non-linearity of age effect by introducing a quadratic term, but it was never significant, therefore not reported. In all analyses, TL, dROMs and OXY were log transformed for normality.

To evaluate effects of brood size manipulation on nestling growth, we also used a repeated measure LMM with body mass as a dependent variable, while time (points at which nestling body mass measurements were taken, Fig. S1) and experimental treatment were fixed factors. Here, apart from individual id, due to the cross-fostering procedure, we considered two additional random parameters: the identity of a nest of rearing (nested within a pair of nests) to control for any effect of shared environment and a nest of origin to account for the fact that some individuals raised by different parents were biological siblings. We tested nestling survival using a binominal GLMM (including brood size treatment as a fixed factor and the abovementioned random factors). We included donor nestlings raised in experimental nests in all these analyses. All analyses were performed in IBM SPSS 23 (IBM Corp. Armonk, NY).

## Results

Mean ± SD parental age (in days) at mating did not differ between the treatments (enlarged 199 ± 17.0 vs control 194 ± 17.4) and sexes (males 197 ± 17.6 vs females 195 ± 17.2). TL did not differ between the experimental groups and sexes prior to the treatment and it was not dependent on individual age. Initial body mass, dROMs and OXY levels were uniform between the treatments and sexes and age-independent (Table S1).

### Effects of the brood size manipulation on nestlings

Number of fledglings significantly differed between enlarged and control nests (median brood size at 65 days post-hatch: 6 vs 4; Mann-Whitney *U* = 62.0, *N* = 36, *P* = 0.001), whereas initial brood size (number of hatchlings) before the treatment did not differ between enlarged and control nests (median brood size: 5 vs 5; Mann-Whitney *U* = 126.0, *N* = 36, *P* = 0.211). Brood size enlargement negatively affected nestling growth rate (Fig. S1). Nestlings from enlarged broods had significantly lower body mass starting from 12 days of age (this effect persisted after independence, i.e. 65 days after hatching) whereas the difference between the treatments in tarsus length at 30 days post-hatch was not significant (Table S2, Fig. S1). The total mean ± SD mass of nestlings reared in an enlarged nest was ca. 40 % higher than in a control nest (at 12 days of life, in grammes: 76.3 ± 9.14 vs 54.6 ± 8.30; *t* = −7.472, *df* = 34, *P* < 0.001). The energetic content of a nestling is positively correlated with nestling mass (Lemon 1993), and daily parental energy expenditure is positively associated with number of young added in an experimental brood size manipulation (Deerenberg et al. 1995). This suggests that our manipulation was effective and as such can be considered as a reliable approximation of higher reproductive effort among parents attending enlarged broods (Santos and Nakagawa 2012). Despite the lower nestling body mass in enlarged broods, post-fledging survival (until 250 days of life) was uniform between groups (*F*_1,237_ = 1.076, *P* = 0.301).

### Effect of the brood size manipulation on parental mass

**Fig. 2:**
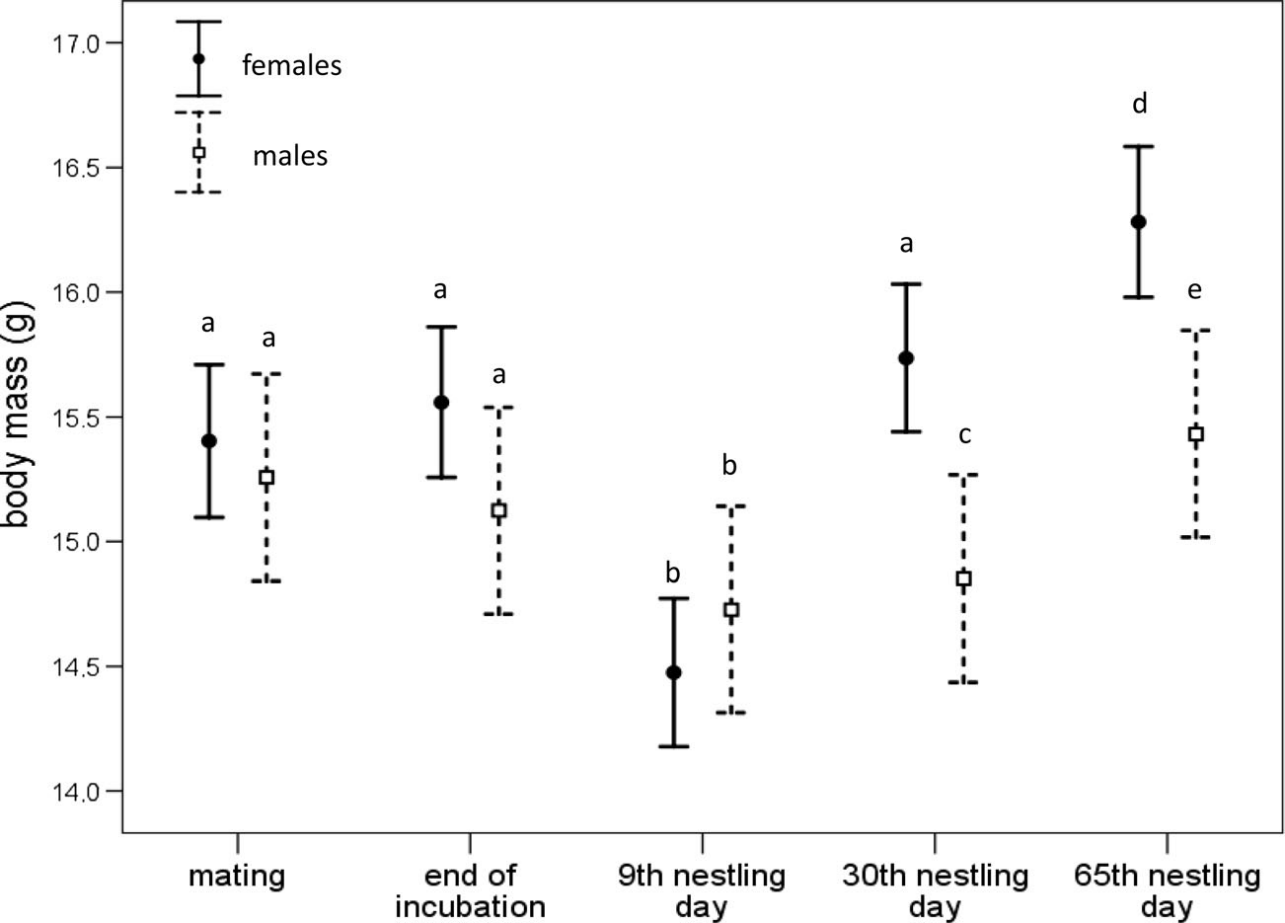
Parental mass throughout duration of the experiment according to sex. Repeated measures for *N* = 36 males and *N* = 36 females. Means that do not share the same letter are significantly different from each other (post hoc LSD, *P* < 0.05). Least square means ±95%CI are shown

Parental body mass was not affected by the experimental treatment but varied with time. Body mass significantly dropped during the highest feeding effort and was regained after reproduction (post hoc LSD both *P* < 0.001; Table S1, Fig. 2). The mass loss appears to be more pronounced among females than among males during the reproductive cycle as indicated by the significant sex-by-time interaction (Table S1), but females also regained more mass after reproduction (at 30 and 65 days after hatching of their young). As a result, females were heavier than males at both these time points (post hoc LSD both *P* = 0.001).

### Effects of the brood size manipulation on TL, oxidative stress parameters and survival of the parents

**Fig. 3:**
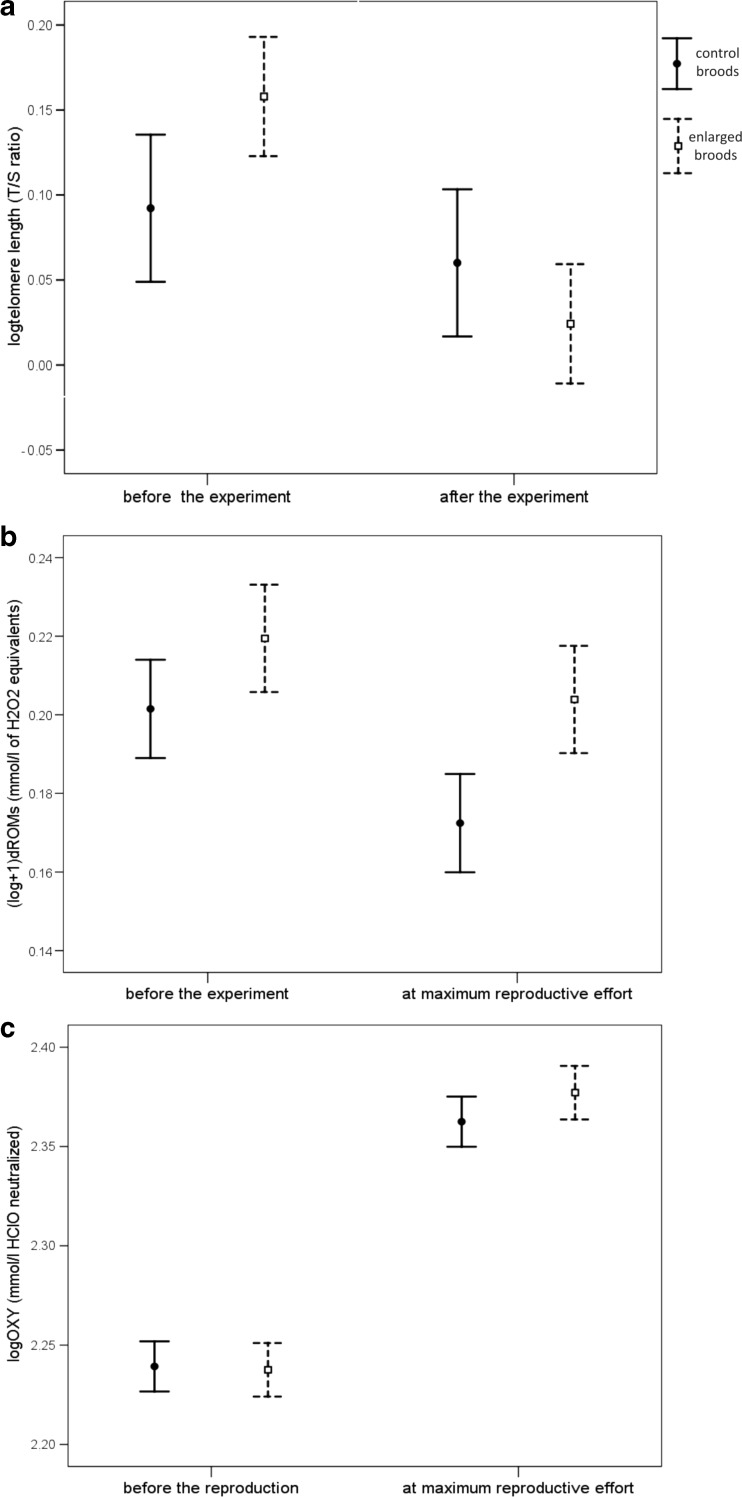
Telomere length, oxidative damage compounds and antioxidant capacity according to time and experimental treatment in adult zebra finches. **a** Relative telomere length [the ratio (T/S) of telomere copy number (T) and single control gene copy (S)] in *N* = 35 control parents and *N* = 36 parents attending experimentally enlarged nests. **b** Oxidative damage compounds in *N* = 27 control parents and *N* = 25 parents attending experimentally enlarged nests. **c** Antioxidant capacity in *N* = 31 control parents and *N* = 34 parents attending experimentally enlarged nests. Least square means ±95%CI are shown

We found the treatment-by-time interaction to be not significant in the analyses of TL, dROMs and OXY, which means that the experimental treatment did not affect the dynamics of these parameters (Table 1, Fig. 3a–c). TL and OXY levels appear to change with time, but this effect could not be directly interpreted while the models involve non-significant interactions. Thus, we repeated the analyses after removing the interaction and the treatment effect. The simplified models confirmed that TL shortened significantly between the start and the end of reproduction (*F*_1,70_ = 7.013, *P* = 0.010) while OXY levels were significantly higher when measured at the peak parental effort than before the treatment (*F*_1,64_ = 51.418, *P* < 0.001). dROM levels did not differ between these two points in time (*F*_1,51_ = 2.406, *P* = 0.127), and females exhibited higher levels of dROMs than males (*F*_1,16.5_ = 5.883, *P* = 0.027). We also tested if the dynamics of TL and oxidative parameters with time were sex-dependent, but all sex-by-time interactions were not significant, therefore not reported.

**Table 1:** Results of linear mixed models analysing variation in telomere length, oxidative damage compounds and antioxidant capacity in response to the brood size manipulation (enlarged vs control non-manipulated) in adult zebra finches

Variables		Estimate ± SE	*df*	*F* value	*P* value
Telomere length (TL)	Fixed effects				
	Brood size manipulation	0.04 ± 0.06	1, 33	0.087	0.770
	Sex	−0.03 ± 0.04	1, 34	0.406	0.528
	Time	0.13 ± 0.04	1, 69	7.054	*0*.*010*
	Brood size manipulation × time	−0.10 ± 0.06	1, 69	2.645	0.108
	Random effects				
	Nest id	0.01 ± 0.01			
	Individual id	0.02 ± 0.01			
Oxidative damage compounds (dROMs)	Fixed effects				
	Brood size manipulation	−0.03 ± 0.03	1, 21	1.231	0.280
	Sex	0.04 ± 0.02	1, 15	5.214	*0*.*037*
	Time	0.02 ± 0.02	1, 50	2.311	0.135
	Brood size manipulation × time	0.01 ± 0.03	1, 50	0.212	0.647
	Random effects				
	Nest id	0.002 ± 0.002			
	Individual id	0.001 ± 0.002			
Antioxidant capacity (OXY)	Fixed effects				
	Brood size manipulation	−0.02 ± 0.03	1, 62	0.073	0.788
	Sex	0.03 ± 0.02	1, 62	1.614	0.209
	Time	−0.14 ± 0.03	1, 63	50.373	*0*.*000*
	Brood size manipulation × time	0.02 ± 0.04	1, 63	0.194	0.661
	Random effect				
	Individual id	0.004 ± 0.002			

Significant terms are shown in italic (*P* < 0.05)

The brood size manipulation did not affect the probability of parental survival until 250 days after the experiment (Table S3).

## Discussion

Our manipulation of brood size appeared effective, as the treatment strongly affected individual and total offspring mass as well as number of fledged young. However, we were not able to find any effects of experimentally elevated reproductive effort on telomere shortening and increased oxidative stress among parents, even in sub-optimal conditions. It appears that parents forced to rear extra nestlings sacrificed offspring condition in favour of their own self-maintenance. Consequently, we were unable to study a mechanistic link potentially mediating the costs experienced by reproducing animals, as these costs were not apparent in our study system.

The cost of reproduction has been frequently studied using brood size manipulations, but this cost has not always been evident (Santos and Nakagawa 2012). In some studies, such costs were confirmed (Daan et al. 1996; Wiersma et al. 2004), while in the others no effects of elevated reproduction on parents were reported (Roulin et al. 1999; Wegmann et al. 2015). In captive zebra finches, brood size manipulation effects are not straightforward as well (Deerenberg et al. 1996; Reichert et al. 2014). The lack of an experimental effect on studied parameters could be theoretically attributed to a life-history strategy adopted by zebra finches. They are opportunistic breeders, can breed whenever the resources are sufficient, and thus might not be willing to work above certain levels to maximize current reproduction. In other words, the brood size manipulation might have not increased actual parental effort. The cost of reproduction may be more apparent in seasonal breeders which are more determined to keep their current reproductive output at higher costs (Piersma 2011). Additionally, our birds were first time breeders, which might have also precluded them from investing in current reproduction in expectation of further breeding events. The negative effects of reproduction on TL and oxidative balance may only be apparent in older birds that have already bred many times. Since ageing-related biomarkers were not altered by elevated reproduction, we did not expect any effect of the treatment on the parental survival rate. Indeed, brood size manipulation did not affect the probability of parental survival until 250 days after the experiment (Table S3).

Despite the fact that we were not able to detect reproductive costs in terms of telomere shortening and increased oxidative stress, we found interesting dynamics of oxidative stress parameters during the course of breeding which might be important for understanding some controversies over the origin of reproductive costs. We detected a significant upregulation of antioxidant capacity during the most demanding reproductive period (Fig. 3c), but the elevation of antioxidant capacity was not dependent upon brood size manipulation. Presumably, it is profitable to pay the cost of antioxidant production in order to avoid oxidative damage at the peak of reproduction. Such a result might contradict the hypothesis that the requirements of reproduction can divert resources from self-maintenance (Monaghan et al. 2009), at least in our study system. Moreover, it appears that ROS may not be directly related to metabolic rate, which is commonly assumed to increase due to reproductive activities (Skibiel et al. 2013; Speakman and Garratt 2014). In fact, Speakman and Garratt (2014) strongly suggest that high metabolic rate may potentially lead to decreased ROS production due to lower mitochondria inner membrane potential and higher activity of uncoupling proteins. Thus, it seems that entering into the reproductive state may augment the defence mechanisms against free radicals, which is in accordance with the oxidative shielding hypothesis (Blount et al. 2015). While interpreting these results, it has to be kept in mind that a dROM test is not a simple measure of ROS production (Costantini and Dell’Omo 2006) since there are several antioxidants already acting before ROM formation. Furthermore, non-enzymatic and enzymatic antioxidants are differentially regulated in zebra finches (Costantini et al. 2011); hence, our quantification of non-enzymatic antioxidants with the OXY test does not represent the entire scope of the antioxidant system.

Our birds experienced a significant drop in body mass during the highest feeding effort, but it was quickly restored after the reproductive effort had ceased (Fig. 2). In this case, birds have to rely on physiological flexibility and optimize their energetic savings, preferably not compromising the most vital biomolecules and functions (Vézina and Salvante 2010). A reduction of oxidative defences may have serious long-term consequences on survival and lifetime reproductive success (Sohal and Weindruch 1996), whereas body condition can be easily rebuilt if resources are available. Lower body mass during peak reproduction could also be perceived not as a cost of reproduction, but as a physiological adaptation to spare energy required for self-maintenance and upregulation of damage-preventing defences (Hillstrom 1995).

We observed TL loss at the end of breeding in all parents irrespective of the treatment (Fig. 3a). This provides additional evidence for within-individual telomere shortening with age. Age-related telomere loss has already been confirmed in longitudinal studies of captive (Heidinger et al. 2012) and natural populations (Asghar et al. 2015). Usually, significant telomere loss is detected in yearly intervals, but here we show that telomere shortening occurred in barely 65 days. We detected a tendency for parents attending experimentally enlarged broods to lose more telomeres than controls, but this effect was not significant. This appears inconsistent with the previous experimental work involving brood size manipulations (Reichert et al. 2014; Sudyka et al. 2014); however, the negative effect of elevated reproduction on TL demonstrated previously was rather small. The power of our repeated measure analysis of TL was 0.743. We suggest that this would be enough for detection of even small effects and our results are likely to be valid.

Our approach does not assess long-term consequences of breeding on oxidative balance and TL, but looks into the most energetically burdensome moment of a reproductive cycle. Oxidative balance is very sensitive and changes rapidly, yet it is often measured after breeding and not at the point of the highest effort, which should be the focal point while assessing the cost of reproduction. Still, we did not find any link between oxidative stress levels at peak reproduction and subsequent telomere dynamics. Oxidative stress parameters could also be evaluated after an experiment, to see if upregulation of defences is persistent. The outcome of our study might also be related to the methods and tissues used. Most studies are restricted to measuring blood markers, which is not surprising considering the greater importance of longitudinal sampling (which controls for individual differences in quality). It has been argued that the positive influence of reproduction on oxidative balance had mostly been detected in tissues such as the liver, kidneys and muscles, while damage in reproducing animals was detected in the blood (Speakman and Garratt 2014). Yet, our results and the previous studies on canaries (Costantini et al. 2014) show that such a stimulating effect can also be confirmed in blood markers.

Our study could add to the debate concerning methodological approaches while assessing reproductive costs. It has been suggested that reproductive effort should be manipulated and not simply compared between breeders and non-breeders. Reproducing animals are a subset of a population of specific quality. Presumably, they have more resources to expend on both oxidative stress management and reproduction; hence, they may allocate available resources differently than non-breeders (Metcalfe and Monaghan 2013). On the other hand, it has been argued that comparing breeders to non-breeders is valid, provided that individuals are randomly allocated to the groups (Speakman and Garratt 2014). Any level of reproduction is costly in terms of metabolic rate while compared to a non-reproducing animal, and this is expected to bring about oxidative costs due to ROS formation. Many studies, however, have shown that breeding animals could actually elevate their oxidative defence mechanisms (Blount et al. 2015) and avoid the consequences potentially leading to reduced survival. Yet again, this notion seems to ignore the fact that metabolic rate could also be tailored to individual quality. For instance, parental daily energy expenditure was positively associated with number of young added in an experimental brood size manipulation, whereas there was no association with an original brood size (Deerenberg et al. 1995). The results of a recent meta-analysis (Blount et al. 2015) also revealed that the mechanisms behind oxidative stress regulation in breeding animals experiencing various levels of reproductive effort are different than when simply compared between breeders and non-breeders. This meta-analysis showed that the positive association between reproductive effort and oxidative damage held only for observational data that did not manipulate reproductive effort. It is hard to determine causality or direction of this effect; for example, individuals that breed may have different pre-reproductive oxidative damage levels than the ones that do not. In fact, it has recently been shown that initial levels of oxidative stress indeed influence reproductive decisions (Costantini et al. 2015). Our study provides more evidence that oxidative balance is differently regulated in animals breeding beyond their chosen rate as we did not detect any association between the level of reproductive effort and oxidative stress.

Due to the low ambient temperature experienced by our birds, the results that we present should not be extended to all breeding situations, but could be applied to populations breeding under harsh conditions or on low-quality territories. Blount et al. (2015) demonstrated that the effect of reproduction on oxidative damage can be driven by the results of laboratory studies, where resources are superabundant. After excluding laboratory studies, there was no significant effect of either reproductive effort or breeding vs non-breeding state on any oxidative stress parameter in any organ whatsoever (Blount et al. 2015, Supplement). This is yet another justification for applying an energetic constraint in our study design. The lack of change in oxidative damage and the increase in antioxidant capacity at the point of the highest reproductive effort, irrespective of brood size manipulation, could not simply be attributed to the fact that low ambient temperature may preclude birds from investing in current reproduction at the benefit of self-maintenance. On the contrary, it has been demonstrated that cold acclimation does not affect plasma antioxidants and oxidative damage in zebra finches (Beamonte-Barrientos and Verhulst 2013) and that plasma antioxidant capacity can even decrease in response to chronic cold exposure (Stier et al. 2014). Hence, there is no evidence that such conditions increase investment in the self in terms of oxidative balance, but still can act as a limiting factor during reproduction since cold exposure is inevitably metabolically costly (Beamonte-Barrientos and Verhulst 2013). However, thermoregulation costs could be partly compensated in the enlarged-brood group due to the extra heat generated by the increased effort and the larger brood size. Without a detailed experimental work, we cannot exclude that this could mitigate reproductive costs.

In conclusion, we were not able to detect the cost of reproduction in terms of oxidative damage and direct telomere erosion in captive, short-lived birds and even in a sub-optimal environment. Thus, it was not possible to study mechanistic explanations of reproductive costs potentially mediated by changes in oxidative balance and telomere dynamics. However, entering into the reproductive state strongly stimulated self-maintenance by increasing antioxidant capacity. Such a repair system appeared to be independent of the level of reproductive effort. Further enquiries on the cost of reproduction in terms of damage to various biomolecules and their protection by antioxidants should involve more direct physiological mechanisms measured throughout the entire reproductive cycle and in a long-term perspective.

## Electronic supplementary material

ESM 1 (PDF 469 kb)
