# Supplementary material for: Elevated reproduction does not affect telomere dynamics and oxidative stress
Source: Behav Ecol Sociobiol. 2016 Oct 7;70(12):2223–33. doi: 10.1007/s00265-016-2226-8 (PMC5102961; doi:10.1007/s00265-016-2226-8)
Supplement: Supplementary file 1 — (PDF 469 kb) [file 265_2016_2226_MOESM1_ESM.pdf]

Online supplement

Elevated reproduction does not affect telomere dynamics and oxidative stress

Behavioral Ecology and Sociobiology 2016

Joanna Sudyka[1], Giulia Casasole, Joanna Rutkowska, Mariusz Cichoń

[1]*Institute of Environmental Sciences, Jagiellonian University, ul. Gronostajowa 7, 30-387 Kraków, Poland, email: joanna.sudyka@uj.edu.pl*

Table S1 Results of linear mixed models analyzing variation in age, body mass, telomere length, oxidative damage compounds and antioxidant levels prior to the brood size manipulation and parental body mass (repeated measure analysis) throughout the experiment in adult zebra finches

Variables		Estimate ± SE	df	F value	P value
**Age at mating**	*Fixed effects*				
	brood size manipulation	-4.72 ± 4.22	1, 34	1.252	0.271
	sex	-1.89 ± 3.95	1, 35	0.228	0.636
	Random effect				
	nest id	19.66 ± 51.58			
**Body mass at mating**	*Fixed effects*				
	brood size manipulation	-0.29 ± 0.35	1, 68	0.706	0.404
	sex	0.17 ± 0.34	1, 68	0.249	0.620
	age	0.01 ± 0.01	1, 68	1.740	0.192
**Initial telomere length**	*Fixed effects*				
	brood size manipulation	-0.06 ± 0.06	1, 32	0.950	0.337
	sex	-0.06 ± 0.05	1, 32	1.889	0.179
	age	0.002 ± 0.002	1, 66	1.343	0.251
	Random effect				
	nest id	0.01 ± 0.01			
**Initial oxidative damage compounds**	*Fixed effects*				
	brood size manipulation	-0.02 ± 0.02	1, 48	1.086	0.303
	sex	0.03 ± 0.02	1, 48	2.172	0.147
	age	<0.001 ± 0.001	1, 48	0.013	0.911
**Initial antioxidant levels**	*Fixed effects*				
	brood size manipulation	0.01 ± 0.03	1, 61	0.027	0.869
	sex	0.02 ± 0.03	1, 61	0.601	0.441
	age	0.001 ± 0.001	1, 61	0.595	0.444
**Body mass**	*Fixed effects*				
	brood size manipulation	-0.25 ± 0.31	1, 69	0.330	0.568
	sex	0.85 ± 0.31	1, 69	2.308	0.133
	time	-0.79 ± 0.20	4, 276	31.271	**0.000**
	sex × time	-1.10 ± 0.23	4, 276	9.079	**0.000**
	brood size manipulation × time	0.39 ± 0.23	4, 276	1.379	0.241
	Random effect				
	individual id	1.24 ± 0.23			

Significant terms are shown in bold (P<0.05)

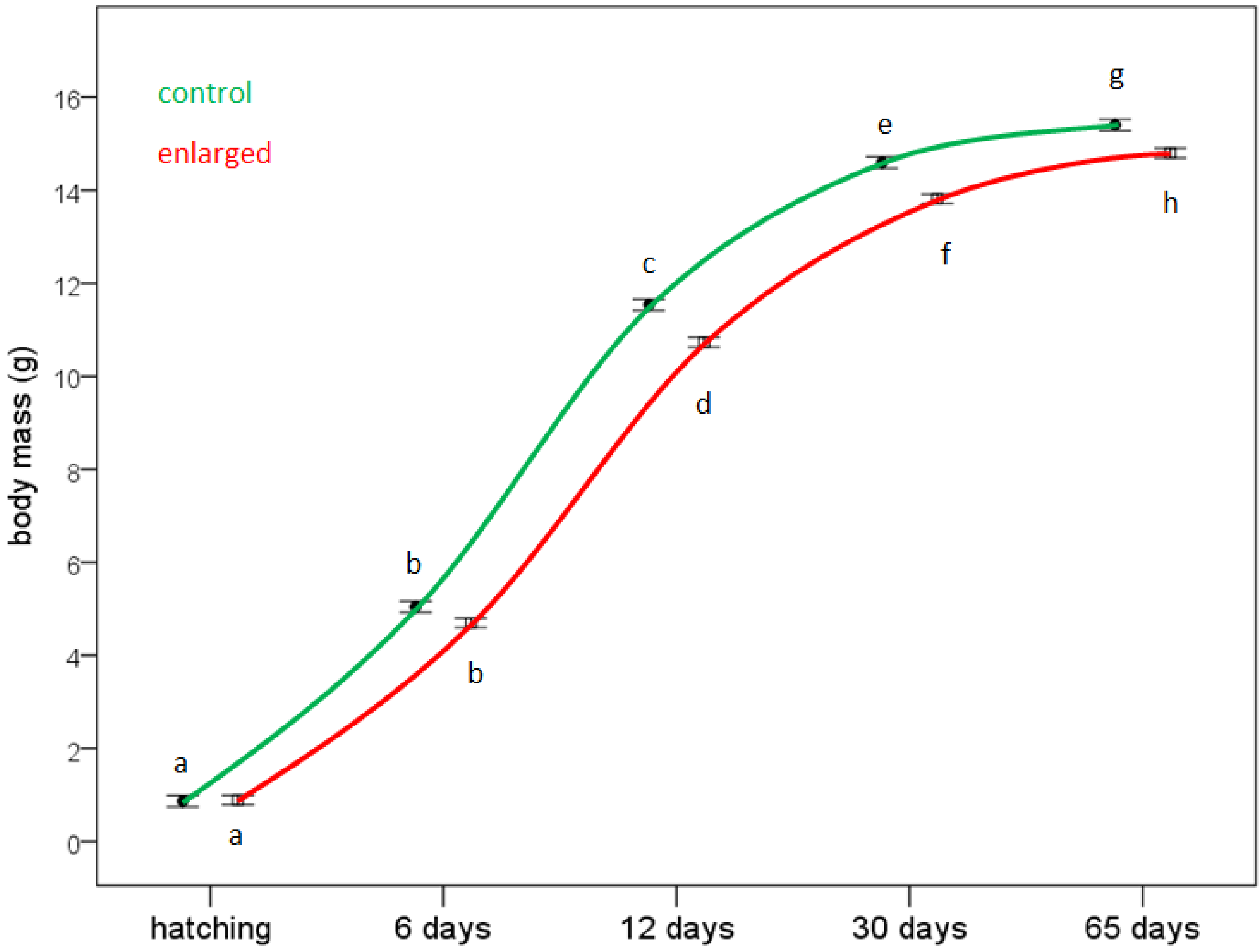

Fig. S1 Nestlings' growth rate according to the treatment. Repeated measures of N = 99 nestlings from control vs N = 141 from enlarged nests. Means that do not share the same letter are significantly different from each other (*post-hoc* LSD, $P < 0.05$). Least square means ± 95%CI are shown.

Table S2 Results of linear mixed models analyzing variation in body mass (repeated measure analysis) and tarsus length at 30 days of age in response to the brood size manipulation (enlarged vs control non-manipulated) in nestling zebra finches

Variables		Estimate ± SE	df	F value	P value
**Body mass**	*Fixed effects*				
	brood size manipulation	0.49 ± 0.20	1, 24	8.870	**0.006**
	time	-13.91 ± 0.14	4, 815	7132.070	**0.000**
	brood size manipulation × time	-0.62 ± 0.21	4, 816	6.034	**0.000**
	Random effects				
	individual id	0.17 ± 0.05			
	nest of rearing (pair)	0.07 ± 0.04			
	nest of origin	0.26 ± 0.08			
**Tarsus**	*Fixed effect*				
	brood size manipulation	0.13 ± 0.09	1, 28	1.923	0.176
	Random effects				
	nest of rearing (pair)	0.03 ± 0.02			
	nest of origin	0.04 ± 0.02			

Significant terms are shown in bold (P<0.05)

Table S3 Results of a binomial generalized mixed model analyzing parental survival until 250 days after the experiment, with the experimental group, sex, age, and the change in telomere length (telomere at 65days of nestling life - telomere before brood size manipulation) as explanatory variables

Fixed effects	Estimate ± SE	df	F value	P value
brood size manipulation	20.706 ± 9250.97	1,67	<0.001	0.998
sex	1.45 ± 0.93	1,67	2.446	0.123
age	0.04 ± 0.03	1,67	2.160	0.146
telomere change	-0.02 ± 0.64	1,67	0.001	0.976
